# Computational Prediction and Analysis of Associations between Small Molecules and Binding-Associated S-Nitrosylation Sites

**DOI:** 10.3390/molecules23040954

**Published:** 2018-04-19

**Authors:** Guohua Huang, Jincheng Li, Chenglin Zhao

**Affiliations:** 1 Provincial Key Laboratory of Informational Service for Rural Area of Southwestern Hunan, Shaoyang University, Shaoyang 422000, China; hnsyljc63@163.com (J.L.); chenglinzhao@126.com (C.Z.); 2 College of Information Engineering, Shaoyang University, Shaoyang 422000, China

**Keywords:** SNO, random forest, fingerprints, information entropy, machine learning

## Abstract

Interactions between drugs and proteins occupy a central position during the process of drug discovery and development. Numerous methods have recently been developed for identifying drug–target interactions, but few have been devoted to finding interactions between post-translationally modified proteins and drugs. We presented a machine learning-based method for identifying associations between small molecules and binding-associated S-nitrosylated (SNO-) proteins. Namely, small molecules were encoded by molecular fingerprint, SNO-proteins were encoded by the information entropy-based method, and the random forest was used to train a classifier. Ten-fold and leave-one-out cross validations achieved, respectively, 0.7235 and 0.7490 of the area under a receiver operating characteristic curve. Computational analysis of similarity suggested that SNO-proteins associated with the same drug shared statistically significant similarity, and vice versa. This method and finding are useful to identify drug–SNO associations and further facilitate the discovery and development of SNO-associated drugs.

## 1. Introduction

Disease is one of the most serious threats to the safety of lives. In 2006 alone, for example, 1,685,210 new cancer cases and 595,690 cancer deaths took place in the United States, and cancer was rising to become the second-leading cause of death [1]. Although very complicated, the cause for this phenomenon was grouped into two main factors. For one thing, the disease’s pathological process is very complex. Environmental, epigenetic, and lifestyle factors jointly determine the development and progress of disease [2]. The cause of the poorly understood Alzheimer’s disease, for example, is believed to be a combination of genetics and history of head injuries, depression, or hypertension [3]. For another, the discovery and development of new drugs could not match the requirement of treating the disease at all, because it is time-consuming and labor-intensive. The cost and the time of developing a new drug was conservatively estimated at an average of more than $ 800 million and at an average of 12 to 15 years, respectively [4]. For drug discovery and development, the first important thing is to find drug targets related to diseases.

Although a large number of computational methods have been proposed to predict drug–target interactions in the past decades [5,6,7,8,9,10,11,12,13,14,15,16,17,18,19], few were dedicated to such specific types of proteins as post-translationally modified proteins and membrane proteins. Because most of the identified targets were not associated with specific diseases, this limited the discovery and development of drugs to a certain extent.

S-nitrosylation (SNO) is a type of post-translational modification where a nitric oxide is covalently attached to a cysteine residue in proteins. In addition to regulation of protein structure and functions [20], SNO plays an important role both in normal health and in a wide range of human diseases [21,22]. For example, aberrant protein SNO was implicated especially in neurodegenerative diseases, including Alzheimer’s and Parkinson’s diseases [23,24,25,26]. Protein SNO was also reported to be associated with some cancers, including lung cancer [27,28]. Accumulating evidence suggested that SNO-protein is a therapeutic target for some degenerative diseases and cancers [27,29]. This provides a new idea to treat these diseases: to interfere with diseases through the specific drugs to mediate relevant SNO proteins. In the paper, we propose a computational method to identify drug–SNO associations.

## 2. Experimental Data

Associations between drugs and binding-associated SNO sites were downloaded from the dbPTM database (http://dbPTM.mbc.nctu.edu.tw/), an experimentally validated post-translational modification (PTM) database [30,31]. The PTM sites whose side chains are located within 10 Å of a drug-binding SNO site were viewed as drug binding-associated PTMs [30]. We collected 147 pairs of associations between drugs and binding-associated SNO sites, including 38 such SNO sites located in 36 proteins and 125 drugs. All these 147 pairs of associations were regarded as positive samples. We randomly paired 38 SNO sites and 125 drugs. Any pairs that were not identical to positive samples served as negative ones, where SNO sites were supposed not to be associated with the corresponding drug. We yielded 478 negative samples, of which 147, along with the 147 positive samples, composed the training set (see Supplementary Material S1). 36 SNO-protein sequences were retrieved from the Uniprot database, a comprehensive repository of protein sequences and function annotation [32,33,34,35,36]. SNO-protein sequences were cut into peptides with 21 amino acid residues and centering the SNO-modified cysteine. If peptides with the SNO-modified cysteine were of less than 21 residues, we added one “X” character or more to reach it.

## 3. Method

**Figure 1:**
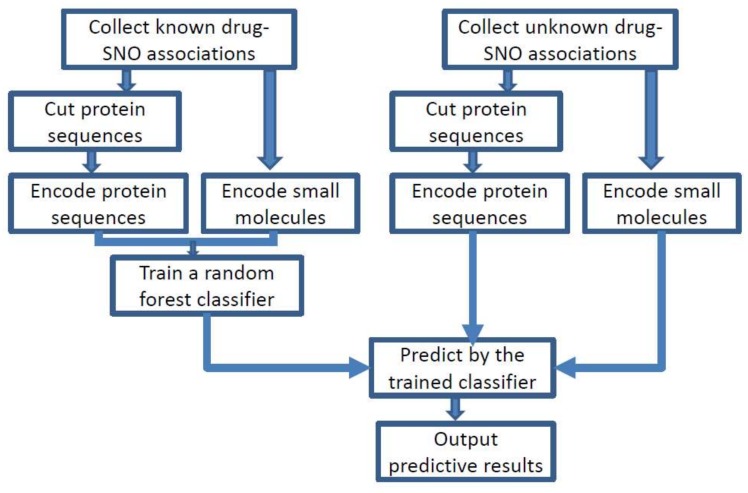
The overview of the proposed method.

As shown in Figure 1, the proposed approach consisted mainly of three steps: encoding, training, and predicting. Peptides and small molecules of drugs were encoded into features by information entropy and molecular fingerprints, respectively. Then, the random forest algorithm [37] was used to train the classifier over the training set. For unknown drug–SNO associations, the trained classifier predicted and outputted the answers “yes” or “no”.

### 3.1. Encoding Small Molecules

**Figure 2:**
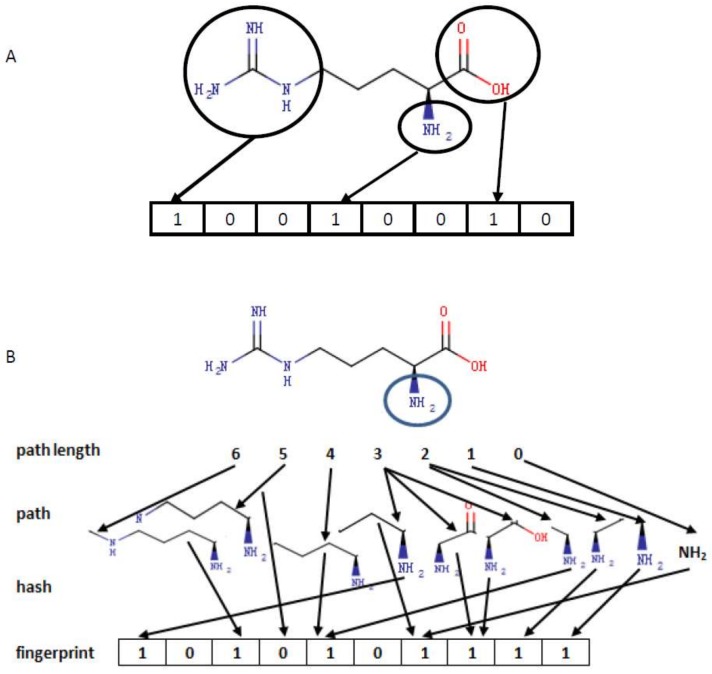
Molecular fingerprints. The top diagram (**A**) illustrates structure-based fingerprints, where eight substructures were explored and three substructures, marked with circles, were present. The bottom diagram (**B**) illustrates hash-based fingerprints, where all paths starting from NH_2_ (circle) and of up to a length of six were explored, and each path was then hashed into a binary bit.

Molecular fingerprinting is an important concept in the area of chemoinformatics and bioinformatics, which has been initiated for chemical substructure search [38], and later, widely used for molecular similarity comparison [39], clustering [40], and so forth. Fingerprinting of small molecules is generally a binary string representation. There are generally two main categories: structure-based and hash-based fingerprints. For the former, each bit represents the presence or absence of a specific substructure; namely, 1 standing for presence and 0 for absence, as illustrated in Figure 2A. Different structure-based fingerprint systems depict different aspects of molecular substructure. Electrotopological state (E-state) fingerprints represent the presence/absence of the 79 E-state substructures defined by Kier and Hall in a molecule [41]. The pubchem fingerprint is an 881-bit binary string, covering a wide range of different substructures and features [42,43]. The MACCS fingerprint [44] depicts 166 key substructures including ISOTOPE, ON(C)C, C$=C($A)$A and C=C(C)C (https://list.indiana.edu/sympa/arc/chminf-l/2007-11/msg00058.html), commonly involving most key chemical features for drug discovery and virtual screening [45]. The chemistry development kit (CDK) substructure fingerprint represents information on the presence of 307 substructures. The CDK extended fingerprint [46] extends the CDK fingerprint with additional bits describing ring features. The CDK graph-only fingerprint is a specialized replacement of the CDK fingerprint which does not consider bond orders [46], and Klekota–Roth fingerprints represent the presence/absence of 4860 substructures. The Daylight fingerprint [45] is a type of hash-based fingerprint, which analyzed the molecular fragments of all paths of up to a user-defined number of bonds, and then hashed every one of these paths, as illustrated in Figure 2B. The CDK Daylight fingerprint hashed each atom type, all augmented atoms, and all paths of 2–7 atoms into a 1024-bit binary string. The CDK hybridization fingerprint is a combination of different fingerprints. All fingerprints mentioned above were calculated by the CDK software [47,48] in the webserver ChemDes, a platform providing multiple software in an online manner for computing molecular descriptors and fingerprints [49]. Table 1 listed these fingerprints and the numbers of bits.

### 3.2. Encoding Protein Peptides

In the field of bioinformatics, there are numerous ways of encoding biological sequences [50,51,52,53,54,55,56] such as the widely used amino acid composition [52], feature vector [53], pseudo amino acid composition [54], and amino acidphysicochemical properties [55], etc. We used the information entropy to encode each peptide. The information entropy of amino acid (*IEA*) was computed by:

(1) $$IEA(\lambda )=\sum_{i=1}^{n}P_{i}(\lambda )\log_{2}P_{i}(\lambda )$$

where λ represents one of 21 kinds of amino acid and $P_{i}(\lambda )$ denotes the probability of occurring at the *i*th position for the amino acid λ. Equation (1) measures the information on the distribution of position for the specific amino acid. Similarly, the information entropy of the position (*IEP*)was denoted by:

(2) $$IEP(i)=\sum_{\lambda \in \Omega }^{}P_{i}(\lambda )\log_{2}P_{i}(\lambda )$$

where Ω denotes the set of 21 amino acids. The IEP measures information on the probability of an amino acid at a specific position. All these 38 protein peptides in the training set were used to estimate the probabilities both of positions for a specific amino acid and of an amino acid at a specific position. For each peptide, the information entropy over all the peptides minus that over the whole set subtracting this peptide was used to encode it. Because all the residues at the 11th position were always cysteine, its information entropy was 0 and was removed. Finally, we obtained 41 features to depict the peptides, each feature ${r^{\prime }}_{i,j}$ normalized by:

(3) $$r_{i,j}=\frac{{r^{\prime }}_{i,j}-\underset{i}{\max}\{{r^{\prime }}_{i,j}\}}{\underset{i}{\max}\{{r^{\prime }}_{i,j}\}-\underset{i}{\min}\{{r^{\prime }}_{i,j}\}},j=1,2,\cdots ,41$$

The 41 features represent 21 and 20 information entropies of amino acids and of position, respectively.

### 3.3. Random Forest

Random forest, which combines bagging and random selection of features, is a type of ensemble learning algorithm [37]. The random forest was described by three key steps: (1) sample with replacement *n* training examples; (2) randomly select *k* features; (3) construct a decision tree using n training examples with *k* features. Repeating the above three steps m times generated m decision trees. For a classification task, the output of testing sample *x* is majority vote of *m* decision trees. Due to advantages such as cheap computation time, ability to deal with high-dimensional data, and better performance, the random forest has been widely applied to classification and regression [57,58,59,60].

#### 3.3.1. Cross Validation and Metrics

We used ten-fold and leave-one-out cross validations to test the proposed method. In the ten-fold cross validation, the training set was randomly grouped into 10 parts of equal or almost-equal size. Each part was in turn taken as testing samples by the trained classifier over the other nine parts. This procedure was repeated ten times. The leave-one-out test is an extreme case of the cross-validation test, where each sample is viewed as an independent part. We used the receiver operating characteristic curve (also known as the ROC curve) to depict experimental performances. The ROC curve could be drawn by plotting the true positive rate against the false positive rate at various thresholds. The area under the ROC curve (AUC) was used to quantitatively assess the experimental performance. The AUC ranges from 0 to 1, with 1 and 0.5 representing the best and uninformative performances, respectively.

#### 3.3.2. Computational Environment

All computations were performed on a Microsoft Windows operating system with a 64-bit version (Windows 10) on an Acer personal computer with an Intel^®^ Core™ i5-3210M CPU and 6.0 G RAM.

## 4. Results and Discussion

**Figure 3:**
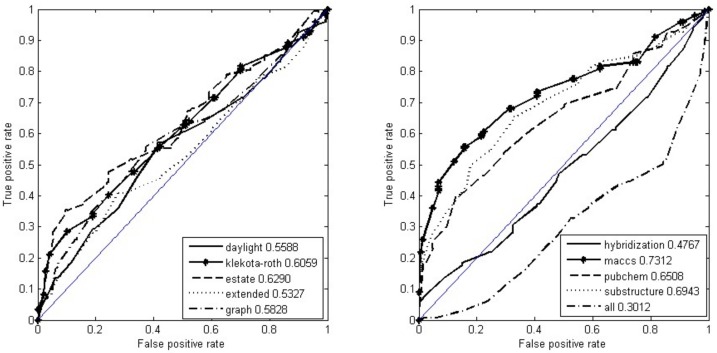
The receiver operating characteristic (ROC) curves of ten types of fingerprint by ten-fold cross validation.

**Figure 4:**
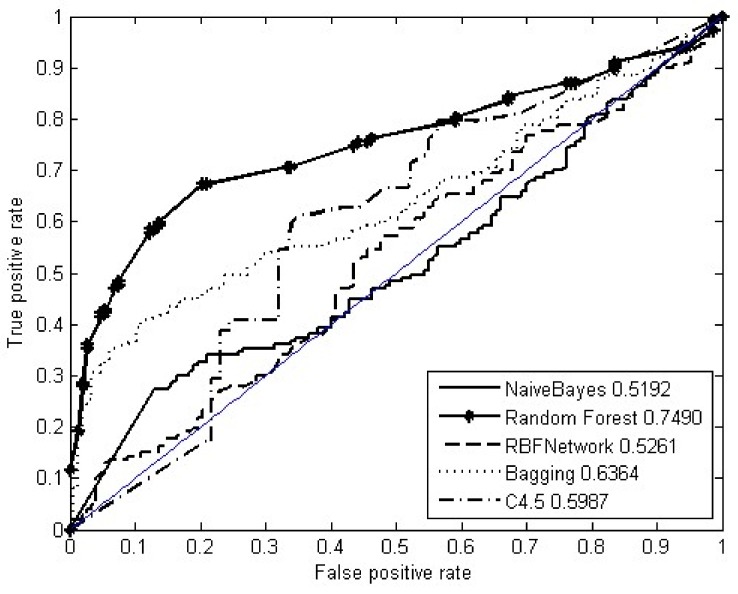
The ROC curves of five algorithms by leave-one-out cross validation.

The ROC curves of the ten-fold cross validation for nine types of fingerprints were drawn in Figure 3. The MACCS fingerprints performed best, followed by substructures, and then by PubChem fingerprints. Their AUCs were 0.7312, 0.6943, and 0.6508, respectively. The combination of nine fingerprints and the hybridization fingerprint performed worst and second-worst, with their AUCs of 0.3012 and 0.4767, respectively. We repeated the ten-fold cross validation ten times. The mean value is 0.7235. Supposing that AUC was the normal distribution with unknown variance values, the 95% confidence interval of the mean AUC was estimated at [0.7160, 0.7311]. To answer the question of if the random forest is a better algorithm for predicting drug–SNO associations, we further compared it with state-of-the-art learning algorithms: C4.5 [61], the naive Bayes classifier [62], the radial basis function network [63], and Bagging [64]. C4.5 is a type of decision tree algorithm [61]. In 2008, C4.5 ranked first in the top 10 data mining algorithms identified by the IEEE International Conference on Data Mining [65]. The naive Bayes classifier [62] is a Bayes’ theorem-based statistical learning model, where variables are supposed to be independent of each other and which makes decisions commonly by computing post-probabilities of samples, given specific classes. The radial basis function network [63] is a specific artificial neural network with radial basis functions as the specific activation function. Bagging [64] is an ensemble learning algorithm, which generally makes decisions by voting over some constituent subclassifiers. Due to excellent performance, these four algorithms have widely been applied to such fields as function approximation and classification. Here, they were used as baseline algorithms for comparison. As shown in Figure 4, the random forest performed best in the leave-one-out cross validation, being 0.1 more than Bagging in terms of AUC.

### 4.1. Computational Analysis of Associations between Drugs and SNO-Proteins

The common hypothesis in the field of molecular biology is the guide by association (GBA) principle [66]. In order to test whether this hypothesis was applicable to associations between drugs and SNO-proteins, we computed the drug–drug and the protein–protein similarities and compared them. Sequence similarity is a basic concept in the field of molecular biology, on which many hypotheses are based. For example, similar sequences are assumed to possess similar structures, which are in turn assumed to have similar functions. We used sequence similarity to measure protein–protein similarity. Each protein sequence was aligned against the whole 38 protein peptides by the PSI-BLAST program [67]. The matrix (*P_i,j_*) _38 × 38_ denoted the alignment score. Each column was normalized by:

(4) $$S(T_{i},T_{j})=\frac{p_{i,j}}{\underset{t}{\max\;}p_{t,j}},i=1,2,\cdots ,38,j=1,2,\cdots ,38$$

To keep the similarity matrix symmetrical, let:

(5) $$PS(T_{i},T_{j})=[S(T_{i},T_{j})+S(T_{j},T_{i})]/2$$

Drug–drug similarity was computed by the Tanimoto coefficient [68], namely:

(6) $$Tc(D_{1},D_{2})=\frac{\left|d_{1}\cap d_{2}\right|}{\left|d_{1}|+|d_{2}|-|d_{1}\cap d_{2}\right|}$$

where |A| denotes the number of shared fingerprints, and $d_{1}$ and $d_{2}$ represent fingerprints of the drugs $D_{1}$ and $D_{2}$, respectively. To keep drug–drug similarity sensible, Tc of less than 0.6 was set to 0.

To test the hypothesis that similar proteins would be associated with similar drugs, we computed similarity $PPS_{}^{d}$ among proteins $\left\{p_{1},p_{2},\cdots p_{k}\right\}$ associated with the same drug by:

(7) $$PPS_{}^{d}=\frac{2}{k(k-1)}\sum_{i=1}^{k-1}\sum_{j=i+1}^{k}TS(p_{i},p_{j})$$

**Figure 5:**
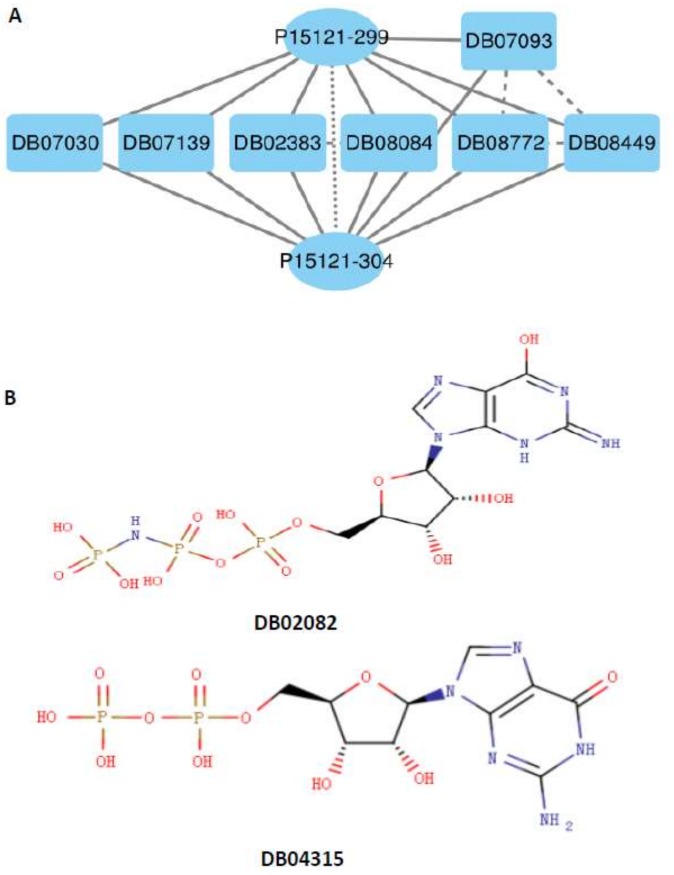
Illustration of cases of similarity. The top diagram (**A**) illustrates the similarity of SNO-proteins associated with the same small molecule. The bottom diagram (**B**) illustrates the similarity of small molecules associated with the same SNO-protein P63000-178.

The similarity $PPS_{c}^{d}$ among other proteins not being associated with the drug d was used as the control. We used a permutation test to examine the above hypothesis. The *p*-value was 0.0020, statistically suggesting that similar SNO-proteins would be associated with the same drugs. Figure 5A demonstrated such a case: two samples P15121-304 and P15121-299 in the protein aldose reductase shared most amino acid peptides, and they were associated with the same drugs: DB02383, DB07030, DB07093, DB07139, DB08084, DB08449, and DB08772 (DBXXXXX is the identifier of a drug in the Drugbank database, which is a unique bioinformatics and cheminformatics resource [69,70]). Similarly, to test hypothesis that similar drugs would be associated with similar SNO-proteins, we computed similarity $\mathrm{DD}S_{}^{p}$ among drugs $\left\{d_{1},d_{2},\cdots d_{k}\right\}$ being associated with the same protein *p* by:

(8) $$DDS_{}^{p}=\frac{2}{k(k-1)}\sum_{i=1}^{k-1}\sum_{j=i+1}^{k}Tc(d_{i},d_{j})$$

The similarity $DDS_{c}^{p}$ among other drugs not being linked to the SNO-protein p was used as the control. The *p*-value in the permutation test is 0.0077, statistically implying that similar drugs would be associated with the same SNO-proteins. As shown in Figure 5B, both drugs phosphoaminophosphonic acid guanylate ester (DB02082) and guanosine-5′-diphosphate (DB04315), which were associated with the same SNO-protein, P63000-178, were very similar in two-dimensional (2D) structure.

### 4.2. Large-Scale Prediction of Unknown Associations of Drugs and SNO-Proteins

In total, 147 of the randomly yielded negative samples in the section “Experimental data” served as negative training samples, and the remaining 331 were used as the testing samples (see Supplementary Materials S2). Of the 331 random associations, 213 were predicted to be negative by the trained classifier over the training set; its accuracy being viewed as 213/331 = 0.6435. Of 118 predicted positive samples, 12 were with the output of probability of 1, implying that they were potential positive samples. We intended to look for evidence to support the prediction by similarity analysis. Next, we investigated SNO-protein targets of small molecules similar to the studied small molecules (*i.e.*, drugs in the column in Table 2). All the small molecules, except DB07905, have similar drugs which all respectively shared the same SNO-protein as the analogue, as shown in Table 2. For example, the association between the small molecule 3-[[*N*-[4-methyl-piperazinyl]carbonyl]-phenylalaninyl-amino]-5-phenyl-pentane-1-sulfonic acid benzyloxy-amide (DB04427) and the tyrosine-protein phosphatase nonreceptor type 1 (P18031) protein was predicted. The small molecule DB04427 was similar to these small molecules: DB06887, DB07719, DB08003, DB08549, DB08591, DB08593, and DB02827, which all targeted the SNO-protein P18031. Thus, it seemed a rational to identify the association of DB04427 with P18031. The protein aldose reductase (P15121) catalyzes the NADPH-dependent reduction of a wide variety of carbonyl-containing compounds to their corresponding alcohols with a broad range of catalytic efficiencies [32,33]. The small molecule 7*N*-methyl-8-hydroguanosine-5′-diphosphate (DB01960) belongs to a type of organic compounds known as purine ribonucleoside diphosphates. The fact that the small molecule DB01960 was similar to the molecules DB02338 and DB03461, which target the SNO-protein P15121, supported predicted DB01960–P15121 associations to a certain extent. The SNO-protein cathepsin K (P43235) was closely involved in osteoclastic bone resorption and may participate partially in the disorder of bone remodeling. The small molecule l-citrulline (DB00155) was predicted to bind the SNO-protein cathepsin K. l-citrulline shares structural similarity with the small molecules DB04276, DB04523, and DB07592, which were associated with P43235. Thus, it seemed a natural inference to predict the DB00155–P43235 association. We indirectly explained the rationality of the above predicted SNO–drug associations in terms of molecular similarity. Due to limitations of conditions, we did not conduct wet experiments to validate these associations, which will be left for experimental biologists to validate in the coming future.

### 4.3. Discussion

For drug discovery and development, it is one of most important things to find protein targets of drugs. Although a large number of approaches have been proposed to detect new targets of drugs in the past decades [5,6,7,10,11,14,71,72,73,74,75,76,77,78], few have dealt with specific proteins such as post-translationally modified proteins which play key regulating roles in cellular activities [79,80]. Protein SNO is involved in the pathological progression of some diseases, especially neurodegenerative diseases. Therefore, identifying associations between SNO-proteins and small molecules is helpful, especially to develop and discover new drugs treating SNO-mediated diseases. For the first time, we have developed a computational method to predict associations between SNO-proteins and small molecules. This method achieved the expected performances on the experimental dataset. We compared the contributions of various fingerprints of molecules to the recognition of associations between drugs and SNO-proteins. Of all the compared fingerprints, the MACCS obtained the best performances (Figure 3), while the combined fingerprints and the hybridization performed worst and second-worst, respectively (Figure 3). Different fingerprints contributed differently, suggesting specificity concerning protein-binding structure. The reason why MACCS fingerprints perform better than the others is unknown, but it is clear that there would be a certain key substructure associated closely with SNO-proteins. Using this fingerprint would promote the performance of predicting associations between drugs and SNO-proteins. Flooding of the informative fingerprints by a large number of irrelative fingerprints would explain why the combination of all fingerprints performed the worst.

In the areas of bioinformatics, there is a common hypothesis called the GBA principle [66]. The computational analysis of drug–drug similarities and protein–protein similarities supported this hypothesis. This provides an idea of finding an SNO-protein-associated drug or a drug-associated SNO-protein from its analogues. However, there are some exceptions observed in the training set. For example, the compounds phosphoaminophosphonic acid guanylate ester (DB02082) and 7*N*-methyl-8-hydroguanosine-5′-diphosphate (DB01960) have a similarity value of 0.8358, but they were associated with different SNO-proteins. Namely, DB01960 is associated with the proteins eukaryotic translation initiation factor 4E type 3 (Q9DBB5), and DB02082 with Ras-related C3 botulinum toxin substrate 1 (P63000). The binding of the drug to the target might be subject to key substructures. Although highly similar, the small molecules DB01960 and DB02082 might lack common key substructures to bind proteins.

## 5. Conclusions

SNO-proteins are involved in the pathological processes of some diseases. There is no doubt that the identification of drug–SNO associations is helpful to discover and develop new drugs to treat SNO-mediated disease. We, for the first time, present a machine learning-based computational method to predict associations between drugs and SNO-proteins. The method is simple to implement but quite efficient. We statistically showed that SNO-proteins associated with the same drug would share high similarity, and vice versa. The finding would be useful in detecting drug-associated SNO-proteins and SNO-protein-associated drugs by the similarity search.

## Figures and Tables

**Table 1:** The categories and the bit numbers of fingerprints.

Category	Number of Bits	Category	Number of Bits
E-state	79	Klekota–Roth	4860
Daylight	1024	MACCs	166
CDK extended	1024	CDK substructure	307
CDK graph	1024	PubChem	881
CDK hybridization	1024		

**Table 2:** Twelve predicted drug–SNO associations, similar drugs, and SNO-proteins targeted by similar drugs.

Predicted Associations	Similar Small Molecules	SNO-Proteins Targeted by Similar Molecules
DB04427–P18031-215	DB06887, DB07719, DB08003, DB08549, DB08591, DB08593, DB02827	P18031-215
DB01960–P15121-299	DB02338, DB03461	P15121-299
DB04315–P15121-299	DB02338, DB03461, DB08772	P15121-299
DB08213–P18031-215	DB02827, DB06887, DB07134, DB07719, DB07730, DB08003, DB08549, DB08591, DB08593	P18031-215
DB04502–P18031-215	DB01962, DB03483, DB03557, DB06887, DB07719, DB08003, DB08549, DB08591	P18031-215
DB00114–P18031-215	DB01962, DB07480	P18031-215
DB02051–P18031-215	DB06887, DB07719, DB08549, DB08591	P18031-215
DB07905–P18031-215	not existing similar drugs	P18031-215
DB08607–P18031-215	DB02072, DB02827, DB03102, DB03670, DB07298	P18031-215
DB02200–P18031-215	DB03483, DB03557, DB03714, DB06887, DB07651, DB07719, DB08003, DB08549, DB08783	P18031-215
DB00171–P15121-299	DB02338, DB03461	P15121-299
DB00155–P43235-139	DB04276, DB04523, DB07592	P43235-139

## Supplementary Materials

The following are available online.

## References

[1] Siegel R.L., Miller K.D., Jemal A. (2016). Cancer statistics, 2016. CA Cancer J. Clin..

[2] Craig J. (2008). Complex Diseases: Research and Applications. Nat. Educ..

[3] Burns A., Iliffe S. (2009). Alzheimer’s disease. BMJ.

[4] Adams C.P., Brantner V.V. (2006). Estimating the cost of new drug development: Is it really $802 million?. Health Aff..

[5] Yamanishi Y., Kotera M., Moriya Y., Sawada R., Kanehisa M., Goto S. (2014). DINIES: Drug-target interaction network inference engine based on supervised analysis. Nucleic Acids Res..

[6] Yamanishi Y., Araki M., Gutteridge A., Honda W., Kanehisa M. (2008). Prediction of drug-target interaction networks from the integration of chemical and genomic spaces. Bioinformatics.

[7] Yamanishi Y., Kotera M., Kanehisa M., Goto S. (2010). Drug-target interaction prediction from chemical, genomic and pharmacological data in an integrated framework. Bioinformatics.

[8] Sawada R., Kotera M., Yamanishi Y. (2014). Benchmarking a Wide Range of Chemical Descriptors for Drug-Target Interaction Prediction Using a Chemogenomic Approach. Mol. Inform..

[9] Chen X., Yan C.C., Zhang X., Zhang X., Dai F., Yin J., Zhang Y. (2016). Drug-target interaction prediction: Databases, web servers and computational models. Brief. Bioinform..

[10] Cheng F., Liu C., Jiang J., Lu W., Li W., Liu G., Zhou W., Huang J., Tang Y. (2012). Prediction of drug-target interactions and drug repositioning via network-based inference. PLoS Comput. Biol..

[11] Chen H., Zhang Z. (2013). A semi-supervised method for drug-target interaction prediction with consistency in networks. PLoS ONE.

[12] Chen X., Liu M.X., Yan G.Y. (2012). Drug-target interaction prediction by random walk on the heterogeneous network. Mol. Biosyst..

[13] Yildirim M.A., Goh K.I., Cusick M.E., Barabasi A.L., Vidal M. (2007). Drug-target network. Nat. Biotechnol..

[14] Huang G., Feng K., Li X., Peng Y. (2016). Large-Scale Prediction of Drug Targets Based on Local and Global Consistency of Chemical-Chemical Networks. Comb. Chem. High Throughput Screen..

[15] Gao Y.F., Chen L., Huang G.H., Zhang T., Feng K.Y., Li H.P., Jiang Y. (2013). Prediction of drugs target groups based on ChEBI ontology. BioMed Res. Int..

[16] Wang Y.C., Zhang C.H., Deng N.Y., Wang Y. (2011). Kernel-based data fusion improves the drug-protein interaction prediction. Comput. Biol. Chem..

[17] Xia Z., Wu L.Y., Zhou X., Wong S.T. (2010). Semi-supervised drug-protein interaction prediction from heterogeneous biological spaces. BMC Syst. Biol..

[18] Vina D., Uriarte E., Orallo F., Gonzalez-Diaz H. (2009). Alignment-free prediction of a drug-target complex network based on parameters of drug connectivity and protein sequence of receptors. Mol. Pharm..

[19] Mei J.P., Kwoh C.K., Yang P., Li X.L., Zheng J. (2013). Drug-target interaction prediction by learning from local information and neighbors. Bioinformatics.

[20] Eichmann C., Tzitzilonis C., Nakamura T., Kwiatkowski W., Maslennikov I., Choe S., Lipton S.A., Riek R. (2016). S-Nitrosylation Induces Structural and Dynamical Changes in a Rhodanese Family Protein. J. Mol. Biol..

[21] Foster M.W., Hess D.T., Stamler J.S. (2009). Protein S-nitrosylation in health and disease: A current perspective. Trends Mol. Med..

[22] Kim J., Won J.S., Singh A.K., Sharma A.K., Singh I. (2014). STAT3 regulation by S-nitrosylation: Implication for inflammatory disease. Antioxid. Redox Signal..

[23] Nakamura T., Tu S., Akhtar M.W., Sunico C.R., Okamoto S., Lipton S.A. (2013). Aberrant protein s-nitrosylation in neurodegenerative diseases. Neuron.

[24] Zahid S., Khan R., Oellerich M., Ahmed N., Asif A.R. (2014). Differential S-nitrosylation of proteins in Alzheimer's disease. Neuroscience.

[25] Nakamura T., Prikhodko O.A., Pirie E., Nagar S., Akhtar M.W., Oh C.K., McKercher S.R., Ambasudhan R., Okamoto S., Lipton S.A. (2015). Aberrant protein S-nitrosylation contributes to the pathophysiology of neurodegenerative diseases. Neurobiol. Dis..

[26] Zhao Q.F., Yu J.T., Tan L. (2015). S-Nitrosylation in Alzheimer’s disease. Mol. Neurobiol..

[27] Wang Z. (2012). Protein S-nitrosylation and cancer. Cancer Lett..

[28] Ben-Lulu S., Ziv T., Weisman-Shomer P., Benhar M. (2017). Nitrosothiol-Trapping-Based Proteomic Analysis of S-Nitrosylation in Human Lung Carcinoma Cells. PLoS ONE.

[29] Nakamura T., Lipton S.A. (2016). Protein S-Nitrosylation as a Therapeutic Target for Neurodegenerative Diseases. Trends Pharmacol. Sci..

[30] Huang K.Y., Su M.G., Kao H.J., Hsieh Y.C., Jhong J.H., Cheng K.H., Huang H.D., Lee T.Y. (2016). dbPTM 2016: 10-year anniversary of a resource for post-translational modification of proteins. Nucleic Acids Res..

[31] Lu C.T., Huang K.Y., Su M.G., Lee T.Y., Bretana N.A., Chang W.C., Chen Y.J., Chen Y.J., Huang H.D. (2013). DbPTM 3.0: An informative resource for investigating substrate site specificity and functional association of protein post-translational modifications. Nucleic Acids Res..

[32] Consortium U. (2014). UniProt: A hub for protein information. Nucleic Acids Res..

[33] Magrane M., Consortium U. (2011). UniProt Knowledgebase: A hub of integrated protein data. Database.

[34] UniProt C. (2010). The Universal Protein Resource (UniProt) in 2010. Nucleic Acids Res..

[35] UniProt C. (2014). Activities at the Universal Protein Resource (UniProt). Nucleic Acids Res..

[36] UniProt C. (2013). Update on activities at the Universal Protein Resource (UniProt) in 2013. Nucleic Acids Res..

[37] Breiman L. (2001). Random forests. MLear.

[38] Christie B.D., Leland B.A., Nourse J.G. (1993). Structure searching in chemical databases by direct lookup methods. J. Chem. Inf. Comput. Sci..

[39] Johnson M.A., Maggiora G.M. (1990). Concepts and Applications of Molecular Similarity.

[40] McGregor M.J., Pallai P.V. (1997). Clustering of large databases of compounds: Using the MDL “keys” as structural descriptors. J. Chem. Inf. Comput. Sci..

[41] Hall L.H., Kier L.B. (1995). Electrotopological state indices for atom types: A novel combination of electronic, topological, and valence state information. J. Chem. Inf. Comput. Sci..

[42] Bolton E.E., Wang Y., Thiessen P.A., Bryant S.H., Wheeler R.A., Spellmeyer D.C. (2008). Chapter 12—PubChem: Integrated Platform of Small Molecules and Biological Activities. Annual Reports in Computational Chemistry.

[43] Chen B., Wild D., Guha R. (2009). PubChem as a source of polypharmacology. J. Chem. Inf. Model..

[44] Durant J.L., Leland B.A., Henry D.R., Nourse J.G. (2002). Reoptimization of MDL Keys for Use in Drug Discovery. J. Chem. Inf. Comput. Sci..

[45] Cereto-Massague A., Ojeda M.J., Valls C., Mulero M., Garcia-Vallve S., Pujadas G. (2015). Molecular fingerprint similarity search in virtual screening. Methods.

[46] Yap C.W. (2011). PaDEL-descriptor: An open source software to calculate molecular descriptors and fingerprints. J. Comput. Chem..

[47] Steinbeck C., Han Y., Kuhn S., Horlacher O., Luttmann E., Willighagen E. (2003). The Chemistry Development Kit (CDK): An open-source Java library for chemo-and bioinformatics. J. Chem. Inf. Comput. Sci..

[48] Steinbeck C., Hoppe C., Kuhn S., Floris M., Guha R., Willighagen E.L. (2006). Recent developments of the chemistry development kit (CDK)—An open-source java library for chemo- and bioinformatics. Curr. Pharm. Des..

[49] Dong J., Cao D.-S., Miao H.-Y., Liu S., Deng B.-C., Yun Y.-H., Wang N.-N., Lu A.-P., Zeng W.-B., Chen A.F. (2015). ChemDes: An integrated web-based platform for molecular descriptor and fingerprint computation. J. Cheminform..

[50] Yu C., Deng M., Zheng L., He R.L., Yang J., Yau S.S. (2014). DFA7, a new method to distinguish between intron-containing and intronless genes. PLoS ONE.

[51] Yu C., Deng M., Cheng S.Y., Yau S.C., He R.L., Yau S.S. (2013). Protein space: A natural method for realizing the nature of protein universe. J. Theor. Biol..

[52] Cai Y.D., Ricardo P.W., Jen C.H., Chou K.C. (2004). Application of SVM to predict membrane protein types. J. Theor. Biol..

[53] Carr K., Murray E., Armah E., He R.L., Yau S.S. (2010). A rapid method for characterization of protein relatedness using feature vectors. PLoS ONE.

[54] Li B.Q., Zhang Y.C., Huang G.H., Cui W.R., Zhang N., Cai Y.D. (2014). Prediction of aptamer-target interacting pairs with pseudo-amino acid composition. PLoS ONE.

[55] Kawashima S., Pokarowski P., Pokarowska M., Kolinski A., Katayama T., Kanehisa M. (2008). AAindex: Amino acid index database, progress report 2008. Nucleic Acids Res..

[56] Tang W., Wan S., Yang Z., Teschendorff A.E., Zou Q. (2018). Tumor origin detection with tissue-specific miRNA and DNA methylation markers. Bioinformatics.

[57] Zhang N., Li B.-Q., Gao S., Ruan J.-S., Cai Y.-D. (2012). Computational prediction and analysis of protein γ-carboxylation sites based on a random forest method. Mol. Biosyst..

[58] Hamby S.E., Hirst J.D. (2008). Prediction of glycosylation sites using random forests. BMC Bioinform..

[59] Ijaz A. (2013). SUMOhunt: Combining Spatial Staging between Lysine and SUMO with Random Forests to Predict SUMOylation. ISRN Bioinform..

[60] Trost B., Kusalik A. (2013). Computational phosphorylation site prediction in plants using random forests and organism-specific instance weights. Bioinformatics.

[61] Quinlan J.R. (1993). C4.5: Programs for Machine Learning.

[62] Russell S., Norvig P. (1995). Artificial Intelligence: A Modern Approach.

[63] Schwenker F., Kestler H.A., Palm G. (2001). Three learning phases for radial-basis-function networks. Neural Netw..

[64] Polikar R. (2006). Ensemble based systems in decision making. IEEE Circuits Syst. Mag..

[65] Wu X., Kumar V., Ross Quinlan J., Ghosh J., Yang Q., Motoda H., McLachlan G.J., Ng A., Liu B., Yu P.S. (2007). Top 10 algorithms in data mining. Knowl. Inf. Syst..

[66] Oliver S. (2000). Proteomics: Guilt-by-association goes global. Nature.

[67] Altschul S.F., Madden T.L., Schaffer A.A., Zhang J., Zhang Z., Miller W., Lipman D.J. (1997). Gapped BLAST and PSI-BLAST: A new generation of protein database search programs. Nucleic Acids Res..

[68] Maggiora G., Vogt M., Stumpfe D., Bajorath J. (2014). Molecular similarity in medicinal chemistry. J. Med. Chem..

[69] Law V., Knox C., Djoumbou Y., Jewison T., Guo A.C., Liu Y., Maciejewski A., Arndt D., Wilson M., Neveu V. (2014). DrugBank 4.0: Shedding new light on drug metabolism. Nucleic Acids Res..

[70] Knox C., Law V., Jewison T., Liu P., Ly S., Frolkis A., Pon A., Banco K., Mak C., Neveu V. (2011). DrugBank 3.0: A comprehensive resource for ‘omics’ research on drugs. Nucleic Acids Res..

[71] Alaimo S., Pulvirenti A., Giugno R., Ferro A. (2013). Drug-target interaction prediction through domain-tuned network-based inference. Bioinformatics.

[72] Gonen M. (2012). Predicting drug-target interactions from chemical and genomic kernels using Bayesian matrix factorization. Bioinformatics.

[73] He Z., Zhang J., Shi X.H., Hu L.L., Kong X., Cai Y.D., Chou K.C. (2010). Predicting drug-target interaction networks based on functional groups and biological features. PLoS ONE.

[74] Wang Y., Zeng J. (2013). Predicting drug-target interactions using restricted Boltzmann machines. Bioinformatics.

[75] Campillos M., Kuhn M., Gavin A.C., Jensen L.J., Bork P. (2008). Drug target identification using side-effect similarity. Science.

[76] Takarabe M., Kotera M., Nishimura Y., Goto S., Yamanishi Y. (2012). Drug target prediction using adverse event report systems: A pharmacogenomic approach. Bioinformatics.

[77] Bleakley K., Yamanishi Y. (2009). Supervised prediction of drug-target interactions using bipartite local models. Bioinformatics.

[78] Dunkel M., Gunther S., Ahmed J., Wittig B., Preissner R. (2008). SuperPred: Drug classification and target prediction. Nucleic Acids Res..

[79] Jia C., Zuo Y., Zou Q. (2018). O-GlcNAcPRED-II: An integrated classification algorithm for identifying O-GlcNAcylation sites based on fuzzy undersampling and a K-means PCA oversampling technique. Bioinformatics.

[80] Wei L., Xing P., Shi G., Ji Z.L., Zou Q. (2018). Fast prediction of protein methylation sites using a sequence-based feature selection technique. IEEE/ACM Trans. Comput. Biol. Bioinform..

